# Impact of antiphospholipid and antinuclear antibodies in coronary artery disease progression

**DOI:** 10.3389/fimmu.2025.1632642

**Published:** 2025-10-16

**Authors:** Tamara Garcia-Camarero, Víctor M. Martínez-Taboada, Juan Irure, Jose M. de la Torre Hernández, Alejandra Comins-Boo, Marcos López-Hoyos, José L. Hernández

**Affiliations:** ^1^ Department of Cardiology, Hospital Marqués de Valdecilla- IDIVAL, Santander, Spain; ^2^ Department of Rheumatology, Hospital Marqués de Valdecilla-IDIVAL, Santander, Spain; ^3^ Departament de Medicina y Psiquiatría, Universidad de Cantabria, Santander, Spain; ^4^ Department of Immunology, Hospital Universitario Marqués de Valdecilla-IDIVAL, Santander, Spain; ^5^ Departamento de Biología Molecular, Universidad de Cantabria, Santander, Spain; ^6^ Department of Internal Medicine, Hospital Marqués de Valdecilla-IDIVAL, Santander, Spain

**Keywords:** coronary artery disease, myocardial infarction, antiphospholipid syndrome, antiphospholipid antibodies, antinuclear antibodies

## Abstract

**Background/Objectives:**

The role of antiphospholipid (aPL) and antinuclear antibodies (ANA) in the progression of coronary artery disease (CAD) remains uncertain. We aimed to determine whether the presence of aPL or ANA predicts CAD progression.

**Methods:**

We conducted a retrospective, single-center, case-control study including patients with CAD classified as either rapid clinical progressors (RCP) or long-standing stable (LSS), and a population-based control group. Autoantibodies analyzed included anticardiolipin (aCL), anti-β2 glycoprotein I (aB2GPI), anti-phosphatidylserine/prothrombin (anti-PS/PT), and ANA.

**Results:**

We included 180 CAD patients (58 RCP, 122 LSS) and 210 matched controls. CAD patients more frequently exhibited positive aCL (p<0.05), whereas aB2GPI IgA was higher among controls. The only significant difference between RCP and LSS was an increased prevalence of aCL IgA in RCP (p<0.05). No consistent differences were found in ANA positivity, antibody subtypes, or overall autoantibody load between groups.

**Conclusions:**

This study does not support a significant role for aPL or ANA in the development or progression of CAD. These findings should be interpreted as hypothesis-generating, and larger, prospective multicenter studies with repeated antibody measurements are required to clarify these associations.

## 1 Introduction

Antiphospholipid syndrome (APS) is an autoimmune disease characterized by thrombotic and/or obstetric events, associated with antiphospholipid antibodies (aPL) (1). Diagnosing APS requires both clinical and serological criteria. Still, patients who do not strictly meet the classification criteria may present with what have been called “clinical manifestations related to APS” or with an inconclusive serological profile not included within the criteria definition (2). Although this concept is applicable in obstetric APS, the available information regarding thrombotic disease, particularly cardiac involvement in APS, is much more limited.

In APS, the heart can be involved through immune-mediated and/or thrombotic mechanisms, and mortality due to cardiovascular disorders is increased (3). Cardiac features of APS include valve abnormalities, coronary artery disease (CAD), myocardial dysfunction, pulmonary hypertension, and intracardiac thrombi (4). aPL may also have a direct role in the atherosclerotic process by inducing endothelial activation, and several traditional and autoimmune-inflammatory risk factors are involved in triggering an expedited atherosclerotic arterial disease in APS (5).

Myocardial infarction (MI) has been reported in 1–5.6% of APS patients, and can be the initial manifestation of the disease, frequently presenting as MI with non-obstructive coronary arteries (3, 6, 7). Other forms of CAD, like unstable angina, have also been associated with APS (8). Coronary artery bypass graft (CABG) in patients with connective tissue disorders (CTDs) provides acceptable outcomes. However, subgroup analysis indicates that patients with APS had significantly increased odds of mortality and a higher rate of complications (9). While there is evidence of the importance of APS in the development of CAD in young individuals (10, 11), its role in an older population is less clear.

Furthermore, some APS-related cardiac manifestations were associated with certain aPL types and/or titer levels. Age and smoking were independent risk predictors for MI in APS, with a significant risk related to lupus anticoagulant (LA) positivity (12). Although APS is more frequent in women, the main thrombotic cardiac manifestation, acute MI, is more frequent in men and particularly in patients with high titers or triple positivity for aPLs (13). Furthermore, microthrombotic/inflammatory myocardial involvement might be subclinical, presenting as diastolic dysfunction, and silent myocardial ischemia and fibrosis detected by imaging techniques are frequently detected in APS patients (14).

A recent scoping review has analyzed the potential role of antinuclear antibodies (ANA) as an independent risk factor for CAD, different from traditional cardiovascular risk factors. A significant positive association was observed between ANA titers and the number of stenotic coronary vessels, particularly in patients with coronary artery ectasia, suggesting that positive ANA could be an independent risk factor for CAD, particularly in individuals without established autoimmune disease (15). Moreover, emerging evidence also suggests that anti-Ro and anti-La antibodies, traditionally linked to autoimmune diseases, may also contribute to the pathogenesis of CAD (16).

Patients with clinically evident CAD differ widely in the rate of progression, with progression being one of the most important factors influencing prognosis. A recent paper, published by our research group, has suggested the existence of specific baseline phenotypic and genotypic markers (inflammatory markers and lipoprotein metabolism) associated with the rapid progression of coronary artery disease (17).

The present study aims to assess the impact of aPL and ANA on CAD in the general population and determine their possible role in differentiating those patients with a rapid clinical progression (RCP) from those with long-standing stable (LSS) disease, an issue that, to our knowledge, has not been previously addressed.

## 2 Subjects and methods

### 2.1 Research design and subjects

We designed a case-control study to assess the impact of aPL on CAD in the general population. Cases were selected from the RAPROMS study (Rapid clinical progressor patient as an emerging clinical entity in patients with coronary atherosclerosis. Exploratory study on possible molecular substrates), a hospital-based single-center retrospective case-control study designed to assess and compare the molecular pattern of several agents involved in the inflammation pathway or lipoproteins metabolism in patients with coronary atherosclerosis (17). This study encompasses two groups of patients: those with RCP of coronary atherosclerosis and those with long-standing stable (LSS) disease.

The control group includes subjects who were taking part in a prospective population-based cohort, the Camargo cohort set up with postmenopausal women and men aged 50 years or older who attended a primary care center in Northern Spain for medical reasons or for their regular health examination, whichever happened first. Full details of this cohort have been previously reported (18–20). Exclusion criteria for controls were the presence of any thrombotic, hematological, connective tissue disorder, or active neoplasia.

This study was designed, implemented, and notified according to the ethical principles established in the Declaration of Helsinki. The information collected from individual cases was completely anonymized and the study was approved by the Ethics Committee of Cantabria (internal code:2017.222).

### 2.2 Definitions of CAD patients’ subgroups

Patients with RCP met the following criteria: a) living patients with at least one coronary artery lesion treated with percutaneous coronary intervention (PCI), and b) with at least two more PCIs in the following 10 years after the index procedure, due to disease progression (excluding restenosis) confirmed on angiography. LSS participants fulfill all of the following criteria: a) living patients with at least one coronary artery lesion treated with PCI, b) uneventful cardiac events in the following 10 years after the index PCI, clinically asymptomatic for angina throughout that period, and with negative noninvasive tests for ischemia. Patients from these two groups were matched by age and sex in a 1:2 design (RCP: LSS).

### 2.3 Clinical data collection

Clinical data from all the patients was collected in a computerized database that included information related to cardiovascular risk factors and main comorbidities with a focus on atherosclerotic and thrombotic events as well as connective tissue disorders.

### 2.4 Laboratory studies

Blood samples were drawn from recruited patients at a stable stage. Apart from the specific autoimmune analysis described thereafter, glucose levels, glomerular filtrate, C reactive protein, and a complete lipid profile were also obtained.

The presence of the following antibodies and aPL isotypes was quantified by commercial enzyme immunoassay in solid phase (ELISA; Biosystems, Barcelona, Spain): anticardiolipin antibodies (aCL) and anti-beta2 glycoprotein I antibodies (AB2GPI) of the IgG and IgM isotypes. The results are reported as quantitative and semiquantitative values. Thus, aCL are quantified in GPL (aCL IgG) or MPL (aCL IgM) according to the standard curve constructed in each test with 5 dilution points of the Harris/Sapporo standards. AB2GPI are quantified as U/ml. Only medium-high titers of aPL were considered positive. Anti-phosphatidyl serine/prothrombin (Anti-PS/PT) antibodies (IgG and IgM isotypes) and IgA aCL and anti-B2GPI were measured by enzyme-linked immunosorbent assay (ELISA) using QUANTA Lite anti-PS/PT and QUANTA Lite B2GPI IgA (Werfen, San Diego, CA, USA). The cut-off value for anti-PS/PT antibodies was established in 30 Units. Results for aCL y anti-B2GPI IgA were set as follows: negative, <20 units; low-medium, 20-80 units; high, >80. Lupus anticoagulant was not determined in the present study.

At baseline, serum samples from each subject were obtained and used for ANA testing. Specifically, ANA was determined by indirect immunofluorescence (IIF) on HEp-2 cells (Biosystems, Barcelona, Spain), and anti-dsDNA and anti-ENA were detected using “Aptiva CTD Essential” (Werfen, Barcelona, Spain).

- *IIF assay on HEp-2 cells*: Sera were diluted 1:160 with phosphate-buffered saline (PBS), which was considered the screening dilution. A cut-off 1:160 ANA titer was selected instead of 1:80 to obtain a high specificity (86.2% (CI 95% 80.4–90.5)) maintaining a relatively high sensitivity (95.8% -CI 95% 94.1–97.1-) (21). A Zeiss fluorescence microscope with incident mercury light illumination and filters for activation/emission of fluorescein isothiocyanate (FITC) was used. Slides with fixed HEp-2 cells served as a source of antigens (Biosystems, Barcelona, Spain). FITC-conjugated rabbit anti-human IgG was used as the secondary antibody (Biosystems, Barcelona, Spain). Incubations, washing steps, and mounting microscope slides were done following the manufacturer’s instructions. The slides were inspected under the fluorescence microscope at 40x magnification. Nuclear, cytoplasmic, and mitotic HEp-2 patterns were considered, and the nomenclature for ANA detected using IIF assay on HEp-2 cells was performed according to the International Consensus on ANA Patterns (ICAP) (22, 23).

- *Aptiva CTD Essential:* It was run on Aptiva System (Werfen) was run on Aptiva system (Werfen) based on particle-based multi-analyte technology (PMAT) to simultaneously detect multiple autoantibodies in one single step. PMAT technology is based on the use of a mixture of suspended microparticles that have a unique color code, individually coated with a different antigen. Each unique color code allows the identification of the antigens within the process. After incubation with patients’ sera, particles are washed and incubated with anti-human IgG conjugated to phycoerythrin. Finally, after another washing cycle, particles are aligned in a monolayer and analyzed through digital imaging technology using two LEDs. A first red LED is used to identify the analyte, while a second green LED allows the measurement of the fluorescence intensity. The reaction data are captured digitally by a high-resolution charged coupled device (CCD) sensor. The acquired image is subsequently stored in the analyzer database for calculation and release of quantitative results. To verify the correct instrument functionality, the system uses quality control samples that contain antibodies specific to each analyte tested. Specifically, Aptiva CTD Essential reagent allows the identification of autoantibodies against dsDNA, Ro60, Ro52, SS-B, RNP, Sm, Jo-1, Scl-70, Ribo-P, Centromere B, and DFS70. Levels higher than 40 UI/mL for anti-dsDNA and 20 fluorescent units (FLU) for the rest of the autoantibodies were considered positive.

### 2.5 Modified adjusted global antiphospholipid syndrome score

The adjusted global antiphospholipid syndrome score (aGAPSS) is based on a quantitative score and includes a combination of two classic vascular risk factors (hypertension and hyperlipidemia) and three aPL (lupus anticoagulant, anticardiolipin antibodies -ACL- and anti-β2 anti glycoprotein I antibodies -antiβ2GPI) (24). It was originally developed to identify patients with systemic lupus erythematosus (SLE) at greater risk of thrombotic events and/or pregnancy morbidity (25). We recently published its usefulness in predicting obstetric outcomes also in aPL carriers (26), and several reports have addressed its utility in predicting thrombotic events (27, 28). However, to date, the role of aGAPSS in coronary artery disease (CAD) has only been explored in only two cohorts of patients (28, 29). As in the present study, we did not have the determination of LA (due to limited availability of stored plasma samples suitable for coagulation-based assays), we included the aPS/PT antibodies in the modified aGAPSS (mGAPSS).

### 2.6 Statistical analysis

Results were expressed as numbers (percentage), mean±standard deviation (SD) or median and interquartile range (IQR), as appropriate. Missing data were handled by casewise deletion for the variable under analysis. The overall proportion of missing values was <5% across all variables, and no imputation methods were applied. Student’s t-test, Mann-Whitney U-test, or one-way ANOVA were used to compare quantitative variables, and Chi-squared or Fisher test, to compare categorical data. A two-tailed *p*-value <0.05 was considered statistically significant in all the calculations that were performed with the IBM SPSS 28.0 software (Armonk, NY: IBM Corp).

## 3 Results

### 3.1 General features of the study cohort

The present study includes 180 patients and 210 controls matched by age and sex (**Table 1**). More than 80% of the participants were men and their average age was in the sixties. As expected, the cases had more cardiovascular risk factors, especially smoking (p<0.05) and dyslipidemia (p<0.05). Approximately 7% of cases had a concomitant connective tissue disease or had suffered another previous thrombotic episode (p<0.05). Fifty-eight of the 180 patients with CDA were considered RCP and 122 LSS. As shown in **Table 1**, no significant differences were found between both groups according to general characteristics, the main cardiovascular risk factors, or associated diseases.

**Table 1:** Demographic characteristics, cardiovascular risk factors, and main comorbidities in the different study groups.

	Controls N=210	Cases N=180	LSS N=122	RCP N=58
Age, (yrs, m ± SD)	64.4 ± 8.5	66.0 ± 9.9	66.5 ± 9.5	65.1 ± 0.5
Sex (% males)	174 (82.9)	154 (85.6)	101 (82.8)	53 (91.4)
IMC, m ± SD	28.8 ± 3.5	28.2 ± 3.9	28.7 ± 3.9	28.9 ± 3.8
Cardiovascular risk factors, N (%)				
Smoking	35 (16.7) ^§^	97 (53.9) ^§^	68 (55.7)	29 (50)
Diabetes	32 (15.2)	25 (13.9)	15 (12.3)	10 (17.2)
High blood pressure	89 (42.4)	87 (48.3)	55 (45.1)	32 (55.2)
Dyslipidemia	66 (31.4) ^§^	90 (50) ^§^	56 (45.9)	34 (58.6)
Obesity	72 (34.3)	62 (35.2)	40 (33.3)	22 (39.3)
Associated diseases, N (%)				
Thrombotic events	0 ^§^	14 (7.8) ^§^	9 (7.4)	5 (8.6)
Connective tissue disorders	0 ^§^	13 (7.2) ^§^	7 (5.7)	6 (10.3)

RCP, rapid clinical progressor; LSS, long-standing stable; ^§^Control vs Cases: p<0.05.

The main laboratory parameters are shown in the **Supplementary Table 1**. Patients with CAD had significantly higher glucose levels, better glomerular filtration rate, a better lipid profile (probably due to more intensive treatment), and higher levels of CRP (p<0.05). When the two patient subgroups were compared, RCP patients had lower glomerular filtration rate, lower HDL levels, and lower CRP levels (p<0.05).

### 3.2 Prevalence and types of aPL

Overall, patients with CAD showed more frequently positive aCL, with no significant differences found for aB2GPI or aPS/PT (**Table 2**). However, when the different aPL isotypes were analyzed, surprisingly the only significant difference was a higher prevalence of aB2GPI IgA in the control group. Although overall all aPL were more frequent in patients with RCP, the difference was only statistically significant for aCL IgA (p<0.05). Furthermore, we did not find statistically significant differences in the aPL load between the study populations (**Supplementary Figure 1**).

**Table 2:** Antiphospholipid antibodies profile in the different study groups.

	Controls N=210	Cases N=180	LSS N=122	RCP N=58
aCL global, N (%)	8 (3.8) ^§^	17 (9.4) ^§^	11 (9)	6 (10.3)
aCL IgM, N (%)	6 (2.9)	10 (5,6)	7 (5,7)	3 (5,2)
aCL IgG, N (%)	2 (1.0)	5 (2.8)	4 (3.3)	1 (1.7)
aCL IgA, N (%)	0	3 (1.7)	0 ^#^	3 (5,2) ^#^
aB2GPI global, N (%)	34 (16.2)	19 (10.6)	11 (9)	8 (13.8)
aB2GPI IgM, N (%)	8 (3.8)	7 (3.9)	4 (3.3)	3 (5,2)
aB2GPI IgG, N (%)	3 (1.4)	5 (2.8)	5 (4.1)	0
aB2GPI IgA, N (%)	27 (12.9) ^§^	8 (4.4) ^§^	3 (2.5)	5 (8.6)
aPS/PT global, N (%)	24 (11.4)	31 (17.2)	19 (15.6)	12 (20.7)
aPS/PT IgM, N (%)	21 (10)	26 (14.4)	16 (13.1)	10 (17.2)
aPS/PT IgG, N (%)	4 (1.9)	5 (2.8)	3 (2.5)	2 (3.4)

RCP, rapid clinical progressor; LSS, long-standing stable; ^§^Control vs Cases: p<0.05. ^#^RCP vs LSS: p<0.05.

### 3.3 mGAPSS

It has been previously suggested that the aGAPSS could help to identify the risk of CAD in young patients with APS (28). As shown in **Table 3**, the mGAPSS was significantly higher in our cohort of patients with CAD compared to controls (p<0.05). Interestingly enough, patients with RCP also presented a higher mGAPSS than LSS (p<0.05).

**Table 3:** Modified GAPSS (mGAPSS) in the different study groups.

	Controls N=210	Cases N=180	LSS N=122	RCP N=58
mGAPSS, median [IQR]	2 [0–4] ^§^	3 [1–4] ^§^	3 [1–4] ^#^	4 [3-4.25] ^#^
mGAPSS risk category, N (%)				
<6 (low-risk)	188 (89.5)	149 (82.8)	104 (85.2)	45 (77.6)
6-11 (moderate-risk)	19 (9)	27 (15)	17 (13.9)	10 (17.2)
≥ 12 (high-risk)	3 (1.4)	4 (2.2)	1 (0.8)	3 (5.2)

RCP, rapid clinical progressor; LSS, long-standing stable; ^§^Control vs Cases: p<0.05. ^#^RCP vs LSS: p<0.05. ^§^p for trend, Control vs Cases: 0.06. ^#^p for trend, RCP vs LSS: 0.06.

### 3.4 Prevalence and types of antinuclear antibodies

To further analyze the possible impact of autoantibodies on the development of CAD, in addition to aPL, ANA and their specificities were determined. As previously demonstrated in our setting (29), the frequency of ANA determined by IIF is high, affecting approximately a quarter of individuals over 50. Overall, we did not find significant differences in ANA positivity by IIF between cases and controls (**Table 4**). However, controls had a higher frequency of ANA at a titer of 1/640 than patients with CAD (p<0.05). Likewise, the homogeneous pattern (AC-1 from ICAP classification) was significantly more frequent in controls than in patients (p<0.05). Although overall, ANA were more frequent in patients with RCP, neither the titers nor the ANA patterns allowed them to be distinguished from the LSS subgroup. The specificities of the ANA were analyzed with a particle-based multi-analyte technology (PMAT) using the Aptiva CTD Essential panel, but we did not find statistically significant differences for the different autoantibodies or their load between the study groups (**Supplementary Table 2**).

**Table 4:** Antinuclear antibodies (ANA) in the different study groups.

	Controls N=210	Cases N=180	LSS N=122	RCP N=58
ANA+, N (%)	56 (26.7)	39 (21.7)	24 (19.7)	15 (25.9)
1/160, N (%)	32 (15.2)	27 (15)	18 (14.8)	9 (15,5)
1/320, N (%)	8 (3.8)	9 (5)	5 (4.1)	4 (6.9)
1/640, N (%)	10 (4.8) ^§^	1 (0.6) ^§^	0	1 (1.7)
1/1280, N (%)	5 (2.4)	2 (1.1)	1 (0.8)	1 (1.7)
1/2560, N (%)	1 (0.5)	0	0	0
ANA patterns, N (%)				
Homogenous, N (%)	27 (12.9) ^§^	7 (3.9) ^§^	3 (2.5)	4 (6.9)
Speckled pattern, N (%)	21 (10)	27 (15)	18 (14.8)	9 (15.5)
Nucleolar, N (%)	4 (1.9)	2 (1.1)	1 (0.8)	1 (1.7)
Centromere, N (%)	1 (0.5)	1 (0.6)	1 (0.8)	0
Others, N (%)	3 (1.4)	2 (1.1)	1 (0.8)	1 (1.7)

RCP, rapid clinical progressor; LSS, long-standing stable; ^§^Control vs Cases: p<0.05.

## 4 Discussion

We have presented data from a selected cohort of CAD patients that question the potential role of autoantibodies in general, and aPLs in particular, in the development of CAD and the identification of a subgroup of patients with CAD RCP. In fact, our findings do not support a consistent association between aPL or ANA and CAD progression. While CAD patients overall showed a slightly higher prevalence of aCL, and RCP patients exhibited a higher frequency of aCL IgA, these results were not accompanied by consistent differences in other antibody isotypes or overall antibody load. Conversely, aB2GPI IgA was more prevalent in controls. Taken together, these observations suggest that the markers examined are not reliable predictors of CAD progression.

Although aPLs may play a key role in the development of CAD in young patients (28, 30), their role in older individuals remains controversial (31). In this study, the slight but significant increase in the frequency of aCL in patients with CAD could suggest a possible causal agent or an effect of the inflammatory process itself induced by atherosclerosis. In this context, the fact that the only isotype that differentiates RCP from LSS is aCL IgA supports the latter hypothesis, given that the significance of this isotype has been called into question (32). Conversely, the higher frequency of aβ2GPI IgA in controls would support the hypothesis of a questionable role of aPLs in the development of CAD. Another aspect to consider is the potential of aPLs, in combination with certain cardiovascular risk factors assessed using the mGAPSS. Although our results suggested a higher score among CAD patients, particularly those with rapid progression, we acknowledge that the mGAPPS used in this study constitutes a method that has not been validated in other cohorts and should therefore be considered exploratory and require validation in prospective studies with a larger number of patients.

Recent data suggest that autoantibodies might also be risk factors for the development of cardiovascular disease (33). In this context, various autoantibodies, including rheumatoid factor (31) and ANA (34), have been implicated in the development of cardiovascular disease. Although the presence of ANA, commonly associated with autoimmune diseases, is significantly more prevalent among individuals with severe coronary atherosclerosis than in those with normal coronary arteries (33), other studies have not been able to confirm these observations (31, 35, 36). In the present study, we could not confirm either the increase in the frequency of ANA, as determined by IIF or PMAT, in patients with CAD, or its association with disease severity. The frequency of moderate titers (1/640) was higher in controls than in patients. As we have previously reported, IIF is more sensitive than solid-phase assays. However, the limited specificity of ANA by IIF is clear, since anti-DFS70 antibodies are the most frequent finding in control populations. This highlights their low clinical relevance and lack of association with systemic autoimmune diseases (37). Therefore, ANA positivity should be interpreted with caution, especially in population-based studies.

A population-based study from Israel (16), provides the first evidence of a statistically significant association between anti-Ro/SSA (anti-Ro) and anti-La/SSB (anti-La) seropositivity and CAD. The study retrospectively analyzed data from 17,231 seropositive patients and 84,368 matched controls. The prevalence of CAD was significantly higher among seropositive individuals compared to controls (9.7% vs. 8.1%, OR = 1.23, 95% CI 1.14–1.31, p < 0.001). This association was particularly pronounced in younger individuals, with odds ratios increasing markedly in patients under 40 years of age (OR = 3.36, 95% CI 1.66–6.82, p < 0.001), and remained robust, albeit attenuated, across older age groups. Additionally, the association was stronger in patients with fewer traditional cardiovascular risk factors, suggesting that anti-Ro/La antibodies may contribute to CAD through distinct, inflammation-mediated mechanisms. However, the study acknowledges certain limitations, including reliance on registry-based data, the absence of detailed clinical information, and uncertainty regarding the temporal relationship between seropositivity and the onset of CAD. In contrast with this study, we did not find significant differences in the various specificities studied by PMAT, including anti-SSa and antiSSb antibodies, the autoantibody load, or the association with systemic autoimmune diseases. Our results contribute to this complex and sometimes contradictory field, underscoring the need for larger, well-designed studies that address these questions more definitively.

The present study has several limitations. Firstly, the sample size of this study is not large, although it is similar to other studies addressing rapid angiographic progression and its relationship with various molecular or biological markers (17). Secondly, because this study was retrospective, we missed all the non-survivors who might have been included in the RCP group and who might have had a more aggressive disease presentation. Thirdly, only a single determination of aPL was performed, preventing us from determining whether it was an isolated positivity or if the patients could be classified as APS. In any case, in the prospective phase of this project, those patients with aPL positivity will undergo a new determination 12 weeks after the initial test, and patients will also be tested for lupus anticoagulant. Furthermore, the inclusion of all patients consecutively will allow us to include the most severe subgroup -those who die from vascular causes- and thus address another major limitation of this study. Finally, it would also be valuable to consider that the results may reflect age- and sex-related differences in immune markers, especially given that the cohort is composed predominantly of middle-aged and older men.

## 5 Conclusions

In conclusion, our study does not provide strong evidence supporting a role for aPL or ANA in the development or progression of CAD in a middle-aged and older population. Although some isolated findings were observed, these should be interpreted as hypothesis-generating rather than definitive. Importantly, the absence of significant associations in this cohort does not imply that autoimmunity is irrelevant to CAD. Rather, it suggests that in older individuals, the contribution of autoantibodies may be overshadowed by conventional cardiovascular risk factors. By contrast, autoantibody profiling may prove more relevant in younger patients with premature CAD, in whom immune-mediated mechanisms may play a larger role (38). Future research should therefore prioritize prospective, multicenter studies with repeated antibody measurements, with a particular focus on younger populations, to better delineate the potential contribution of autoimmunity to CAD progression.

## Data Availability

The raw data supporting the conclusions of this article will be made available by the authors, without undue reservation.

## Supplementary material

Supplementary Figure 1 Antiphospholipid antibody load in the different study groups. Antiphospholipid antibody load was assessed in 210 controls and 180 cases (58 rapid clinical progressors (RCP) and 122 long-standing stable disease (LSS)). No significant differences were found between cases and controls, nor between RCP and LSS patients.
