# Supplementary figures and images for: Impact of antiphospholipid and antinuclear antibodies in coronary artery disease progression
Source: Front Immunol. 2025 Oct 16;16:1632642. doi: 10.3389/fimmu.2025.1632642 (PMC12571853; doi:10.3389/fimmu.2025.1632642)

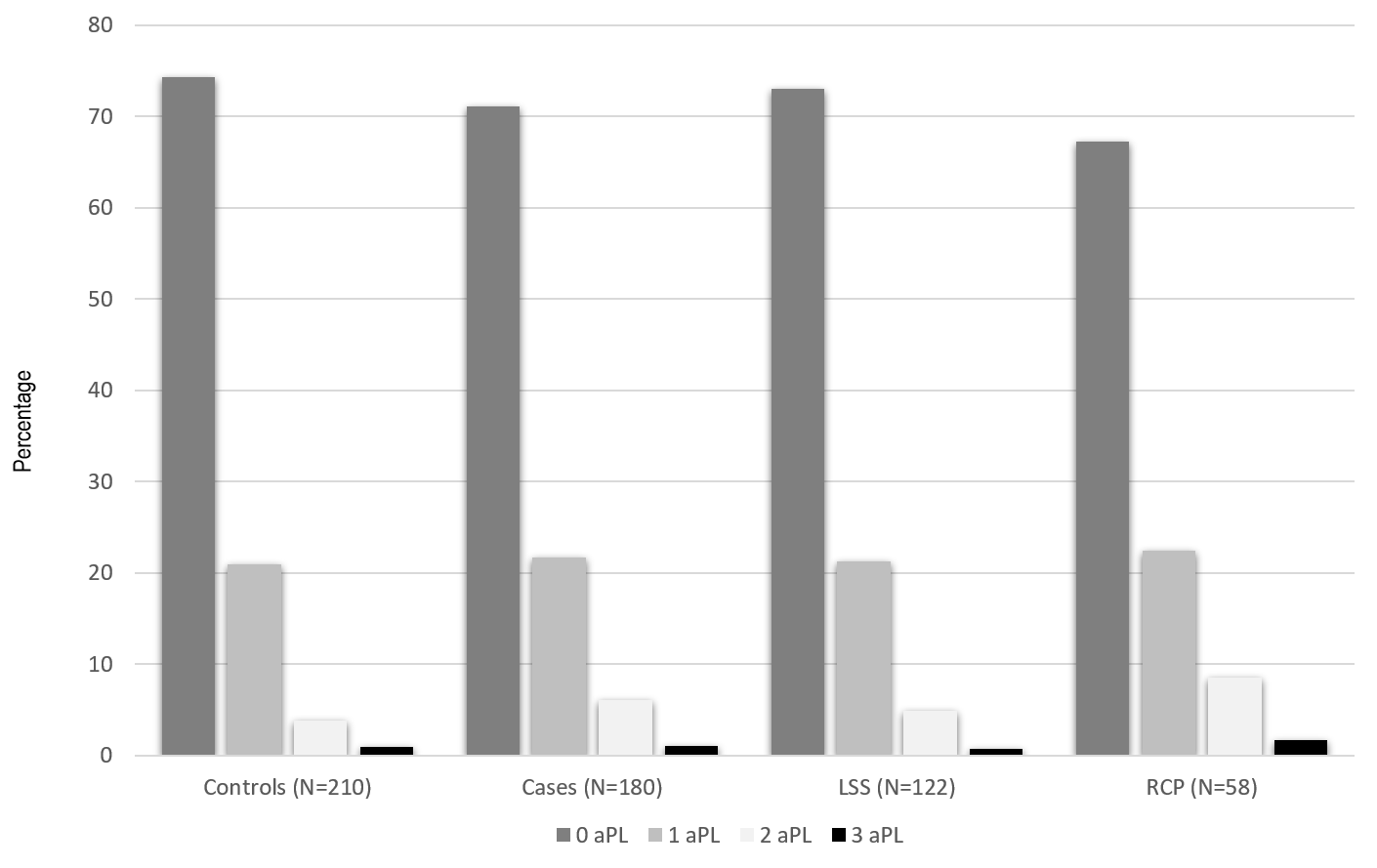

Supplement: Supplementary Figure 1 — Antiphospholipid antibody load in the different study groups. Antiphospholipid antibody load was assessed in 210 controls and 180 cases (58 rapid clinical progressors (RCP) and 122 long-standing stable disease (LSS)). No significant differences were found between cases and controls, nor between RCP and LSS patients. [file Image1.tif]
