# Supplementary material for: Impact of antiphospholipid and antinuclear antibodies in coronary artery disease progression
Source: Front Immunol. 2025 Oct 16;16:1632642. doi: 10.3389/fimmu.2025.1632642 (PMC12571853; doi:10.3389/fimmu.2025.1632642)
Supplement: Supplementary file 2 [file Table1.docx]

**Supplementary Table 1.** Main laboratory parameters in the different study groups.

|  | **Controls**  **N=210** | **Cases**  **N=180** | **LSS**  **N=122** | | **RCP**  **N=58** |
| --- | --- | --- | --- | --- | --- |
| **Glucose,** *m±SD* | 101±31.9 ^§^ | 115.4±36.9 ^§^ | | 113.9±33.8 | 118.2±42.8 |
| **GFR,** *m±SD* | 76.3±17.6 ^§^ | 89.1±28.1 ^§^ | | 92.8±28.8 ^#^ | 81.5±25.2 ^#^ |
| **Total cholesterol,** *m±SD* | 217±39 ^§^ | 153.1±37.9 ^§^ | | 156.1±34.9 | 146.8±43.1 |
| **Tryglicerids,** *m±SD* | 114±57.3 ^§^ | 139.9±79.2 ^§^ | | 139.9±82.9 | 139.8±71.4 |
| **TGC/HDL ratio,** *median [IQR]* | 1.95 [1.31-2.83] ^§^ | 2.09 [1.78-4.84] ^§^ | | 2.82 [1.74-4.77] | 3.36 [1.81-5.03] |
| **HDL,** *median [IQR]* | 52 [45-63] ^§^ | 41 [37-50] ^§^ | | 42 [38-51] ^#^ | 39 [36-45] ^#^ |
| **LDL,** *m±SD* | 137.8±31.9 ^§^ | 82.9±29.3 ^§^ | | 84.7±26.5 | 79.3±34.2 |
| **Cholesterol no HDL,** *m±SD* | 161±35.8 ^§^ | 109.8±34.9 ^§^ | | 111.7±32.1 | 105.9±40.2 |
| **CRP,** *median [IQR]* | 0.2 [0.1-0.5] ^§^ | 0.44 [0.1-1.42] ^§^ | | 0.59 [0.1-1.82] ^#^ | 0.25 [0.09-0.72] ^#^ |

*CRP: C-reactive protein; GFR: Glomerular filtration rate; RCP: rapid clinical progressor; LSS: long-standing stable; ^§^Control vs Cases: p<0.05. ^#^ RCP vs LSS: p<0.05.*
