# Supplementary material for: Impact of antiphospholipid and antinuclear antibodies in coronary artery disease progression
Source: Front Immunol. 2025 Oct 16;16:1632642. doi: 10.3389/fimmu.2025.1632642 (PMC12571853; doi:10.3389/fimmu.2025.1632642)
Supplement: Supplementary file 3 [file Table2.docx]

**Supplementary Table 2.** Aptiva CTD Essential panel in the different study groups.

|  | **Controls**  **N=210** | **Cases**  **N=180** | **LSS**  **N=122** | **RCP**  **N=58** |
| --- | --- | --- | --- | --- |
| **DFS70+,** *N (%)* | 6 (2.9) | 1 (0.6) | 0 | 1 (1.7) |
| **dsDNA+,** *N (%)* | 4 (1.9) | 2 (1.1) | 2 (1.6) | 0 |
| **Jo1+,** *N (%)* | 0 | 0 | 0 | 0 |
| **RiboP+,** *N (%)* | 1 (0.5) | 0 | 0 | 0 |
| **RNP+,** *N (%)* | 1 (0.5) | 0 | 0 | 0 |
| **Ro52+,** *N (%)* | 0 | 0 | 0 | 0 |
| **Ro60+,** *N (%)* | 0 | 0 | 0 | 0 |
| **Scl70+,** *N (%)* | 1 (0.5) | 2 (1.1) | 1 (0.8) | 1 (1.7) |
| **Sm+,** *N (%)* | 0 | 0 | 0 | 0 |
| **SSb+,** *N (%)* | 0 | 0 | 0 | 0 |
| **Centromere,** *N (%)* | 1 (0.5) | 0 | 0 | 0 |
| **CTD antibody load,** *N (%)* |  |  |  |  |
| ***0 antibodies*** | 204 (97.1) | 176 (97.8) | 119 (97.5) | 57 (98.3) |
| ***1 antibodies*** | 5 (2.4) | 4 (2.2) | 3 (2.5) | 1 (1.7) |
| ***≥ 2 antibodies*** | 1 (0.5) | 0 | 0 | 0 |

*RCP: rapid clinical progressor; LSS: long-standing stable*
